# Stimulus-Rate Sensitivity Discerns Area 3b of the Human Primary Somatosensory Cortex

**DOI:** 10.1371/journal.pone.0128462

**Published:** 2015-05-28

**Authors:** Yevhen Hlushchuk, Cristina Simões-Franklin, Cathy Nangini, Riitta Hari

**Affiliations:** 1 Brain Research Unit, Department of Neuroscience and Biomedical Engineering, Aalto University School of Science, 00076 AALTO, Espoo, Finland; 2 Advanced Magnetic Imaging Centre, Aalto NeuroImaging, Aalto University School of Science, 00076 AALTO, Espoo, Finland; Université catholique de Louvain, BELGIUM

## Abstract

Previous studies have shown that the hemodynamic response of the primary somatosensory cortex (SI) to electrical median nerve stimulation doubles in strength when the stimulus rate (SR) increases from 1 to 5 Hz. Here we investigated whether such sensitivity to SR is homogenous within the functionally different subareas of the SI cortex, and whether SR sensitivity would help discern area 3b among the other SI subareas. We acquired 3-tesla functional magnetic resonance imaging (fMRI) data from nine healthy adults who received pneumotactile stimuli in 25-s blocks to three right-hand fingers, either at 1, 4, or 10 Hz. The main contrast (all stimulations pooled vs. baseline), applied to the whole brain, first limited the search to the whole SI cortex. The conjunction of SR-sensitive contrasts [4 Hz − 1 Hz] > 0 and [10 Hz − 1 Hz] > 0 ([4Hz − 1Hz] + [10Hz − 1Hz] > 0), applied to the SI cluster, then revealed an anterior-ventral subcluster that reacted more strongly to both 10-Hz and 4-Hz stimuli than to the 1-Hz stimuli. No other SR-sensitive clusters were found at the group-level in the whole-brain analysis. The site of the SR-sensitive SI subcluster corresponds to the canonical position of area 3b; such differentiation was also possible at the individual level in 5 out of 9 subjects. Thus the SR sensitivity of the BOLD response appears to discern area 3b among other subareas of the human SI cortex.

## Introduction

Tactile input travels from the skin through the thalamus to the primary somatosensory cortex (SI) in the postcentral gyrus. Human SI is not homogeneous but comprises 4 cytoarchitectonically and functionally distinct areas: 3a, 3b, 1, and 2 (see, e.g., [1, 2]). The great inter-individual variability of the cytoarchitectonic SI subareas demonstrated in the above-mentioned studies stresses the need for individual-level noninvasive functional mapping of these SI subareas. Assignment of the observed activations to different subareas of SI in functional magnetic resonance imaging (fMRI) studies has, however, been mainly derived from a rough definition based on macroanatomical landmarks (see, for example, [3, 4]). At the moment of launching this experiment, no studies had demonstrated noninvasive imaging techniques that would reliably differentiate SI subareas at the individual level. Such functional segregation of SI subareas would generate a finer framework for studies of SI cortex, similar to the functional mapping in the studies of the visual cortex [5]. It could also, at least when combined with other structural and functional information available, improve the precision of presurgical mapping, especially in patients with distorted anatomy where macroanatomical landmarks may no longer be reliable.

Here we investigated stimulus-rate (SR) sensitivity of BOLD responses to tactile stimulation to functionally segregate SI subareas. Positron-emission tomography recordings (cf. [6]) and previous fMRI studies [7–10] have demonstrated SR effects in human SI cortex: The measured response at least doubles when the rate of electrical median-nerve stimuli increases from 1 to 4–5 Hz, with less change at SRs above 5 Hz. Consequently, the SR dependence of fMRI responses was suggested as a tool for identifying the human SI cortex among the neighboring cortical areas [7, 8, 10].

In the above studies, the SR effects were evaluated for the whole SI cortex but not at the subarea level. Invasive recordings in primates have, however, demonstrated functional differences between these subareas: Areas 3b and 1 respond primarily to cutaneous stimuli, area 3a mainly receives proprioceptive input, and area 2 processes both tactile and proprioceptive input [11–13]. In humans, cytoarchitectonic population maps demonstrate analogous arrangement of the SI subareas [1], and fMRI has demonstrated similar functional specificity: Area 3a is activated by proprioceptive stimuli, while areas 3b, 1, and 2 are activated by both kinesthetic and tactile stimuli [14]. Hence, by employing tactile stimuli only, one could functionally preclude activation of area 3a and thereby limit the investigation to areas 3b, 1, and 2.

Because of the distinct functional properties of SI subareas, we hypothesized SR sensitivity to vary between areas 3b, 1, and 2. Area 3b mainly receives direct thalamic input, whereas areas 1 and 2 of SI receive their main input from area 3b, with clearly less afferents from the thalamus [15]. Therefore, areas 1 and 2, being downstream from area 3b in the tactile-processing chain [16, 17], reflect more polysynaptic processing than area 3b and are more susceptible to many modulating factors, including the SR. Consequently, response suppression as a function of increasing SR should be stronger in areas 1 and 2 than in area 3b, resulting in relatively stronger responses in area 3b than in areas 1 and 2. Based on changes of the SI hemodynamic responses at SRs from 1 to 10 Hz [6–9], we expected a significant difference between response strengths to 1-Hz vs. 4- and 10-Hz SRs. Therefore, we designed a corresponding SR-sensitive contrast to functionally demarcate the SR-sensitive subarea within the SI cortex—an analysis feature targeting the noninvasive subarea differentiation that previous studies have lacked [6–10, 18].

## Materials and Methods

### Subjects, stimuli, and experimental conditions

We acquired fMRI data from nine healthy adults (6 males, 3 females; mean age 27 years, range 23–33). All subjects gave their informed written consent before the experiment. The experimental protocol had received prior approval by the Ethics Committee of Helsinki and Uusimaa Hospital District.

**Fig 1:**
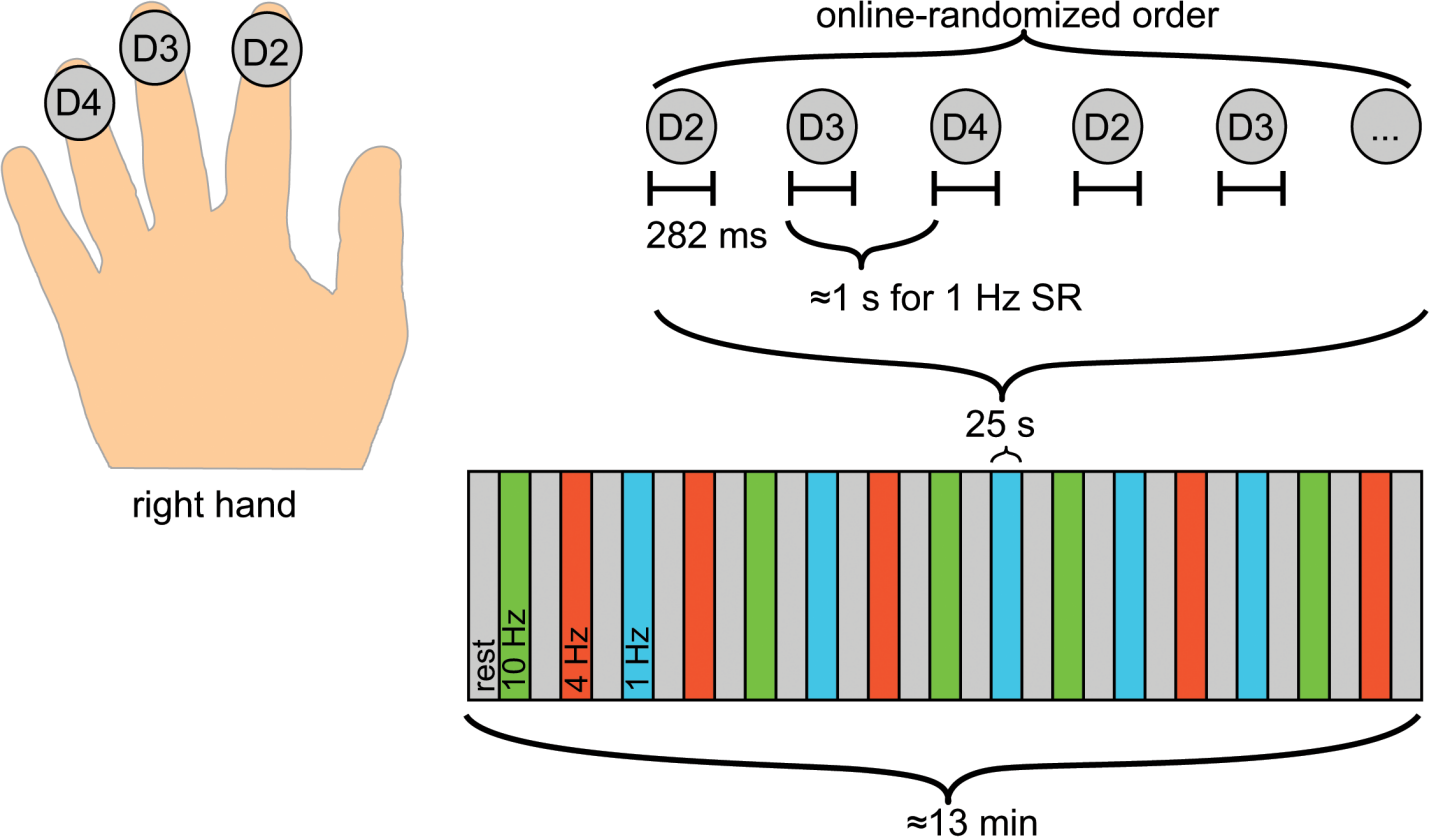
Presentation of the stimuli. The pneumatic tactile stimuli were delivered to the fingertips of the right index (D2), middle (D3) and ring (D4) digits in a randomized order within a stimulation block. 25-s stimulation blocks alternated with the rest blocks of the same duration. While each single stimulus caused deviation of the pneumatic membrane for 282ms, time between onsets of the stimuli corresponded to the stimulus rate (SR) which was fixed (1, 4 or 10 Hz) for each 25-s stimulation block.

Fig 1 illustrates our stimulus paradigm: We presented tactile stimuli in 25-s stimulation blocks. Within each block, the SR was kept constant (1, 4, or 10 Hz resulting in 25, 100 or 250 stimuli in corresponding 25-s blocks) and the tactile stimuli were delivered in a random order to the tips of the index, middle or ring finger of the right hand. The stimuli were produced by balloon diaphragms driven by compressed air [19]. According to pressure measurements, single stimuli lasted for 282 ms (40 ms rise time, 62 ms plateau, 180 ms return to baseline pressure). To avoid tactile adaptation and to give time for the diaphragm to return to the baseline level before the next stimulus, two successive stimuli within a block were never delivered to the same site. We chose to stimulate three different fingers which prevented full predictability of the next stimulus site and increased the average time between the onsets of the stimuli arriving at the same site. Given that three sites were stimulated and no two successive stimuli were delivered to the same site, the diaphragm returned to the baseline level before the next stimulus arrived for 1- and 4-Hz SRs, and for half of the stimuli at 10-Hz SR, resulting in distinct tactile stimuli.

The stimulation and rest blocks lasted for 25 s, and the whole 13-min sequence comprised 5 stimulation blocks per each stimulus rate. The presentation and timing of the stimuli were controlled by a personal computer running Windows 98 and Presentation software (Version 0.60, Neurobehavioral Systems Inc., Albany, CA).

### MRI data acquisition and analysis

We acquired functional MRI data on a Signa 3T MR scanner (GE Medical systems) using a gradient-echo planar imaging sequence with the following parameters: flip angle = 90°, repetition time = 2500 ms, echo time = 32 ms, field of view = 200 mm, matrix 64 × 64, slice thickness 4 mm (resulting in 3.13 × 3.13 × 4 mm^3^ voxels), number of excitations = 1, altogether 31 axial-oblique slices and interleaved slice acquisition. Subsequent analysis excluded the first four (of 314) time points in each slice due to the partial magnetic saturation of the volumes.

Anatomical brain images were obtained in the sagittal plane with a 3-D fast spoiled gradient echo sequence (inversion-recovery prepared): flip angle = 15°, repetition time = 9 ms, echo time = 1.9 ms, field of view = 240–260 mm, matrix 256 × 256, and slice thickness 1.3 or 1.4 mm (resulting in (0.94–1.02) × (0.94–1.02) × 1.3 (or 1.4) mm^3^ voxels).

Preprocessing of the data in BrainVoyager QX (BV QX) software (Brain Innovation B.V., Maastricht, Netherlands) included 3D motion correction, high-pass filtering and linear trend removal, slice scan-time correction, Gaussian spatial smoothing (full width-at-half-maximum = 6 mm), and normalization to Talairach space ([20]; normalized voxel size 2 × 2 × 2 mm^3^). The initial automatic coregistration of the functional data to the anatomical volumes was visually verified and additionally fine-tuned by manually adjusting the linear-transformation values to ensure proper coregistration in the region of the left SI cortex.The predictors for the general linear model were obtained by convolving the box-car time courses of the stimulation blocks with the hemodynamic response function [21]. In addition to these three predictors, the model incorporated 6 movement regressors of no interest obtained during the motion correction. For statistical inferences at the group level, we employed random-effects analysis. To correct for multiple comparisons in the whole-brain analysis, all obtained statistical maps were thresholded using false discovery rate (FDR) at the value q(FDR) < 0.1 [22].

A more detailed description of the methods can be found in our previous report on a negative blood-oxygenation-level-dependent (BOLD) response in the ipsilateral SI cortex, where these data were partially presented (Experiment 1 in [18]).

### Main contrast and stimulus-rate sensitive contrast

All three SRs were pooled together in the main contrast ([10 Hz + 4 Hz + 1 Hz] > 0 corresponding to the contrast vector [10 Hz, 4 Hz, 1 Hz, baseline] = [1 1 1 0]) which revealed clusters activated by all tactile stimuli and, thus, allowed us to isolate the SI cortex. To detect SR-sensitive subclusters within SI, we employed the conjunction of the contrasts: [4 Hz − 1 Hz] > 0 and [10 Hz − 1 Hz] > 0 to search for voxels within the SI cluster in which the responses to both 4-Hz and 10-Hz stimuli were larger than that to the 1-Hz stimuli. The statistical threshold applied within the SI cluster was corrected for multiple comparisons, (q(FDR) < 0.1; for FDR thresholding in small ROIs, see [22]), in the group-level analysis and in the subsequent post-hoc analysis at the individual level. For better spatial specificity, the individual analyses were performed on non-smoothed functional data.

## Results

### Identifying the SI cortex

**Fig 2:**
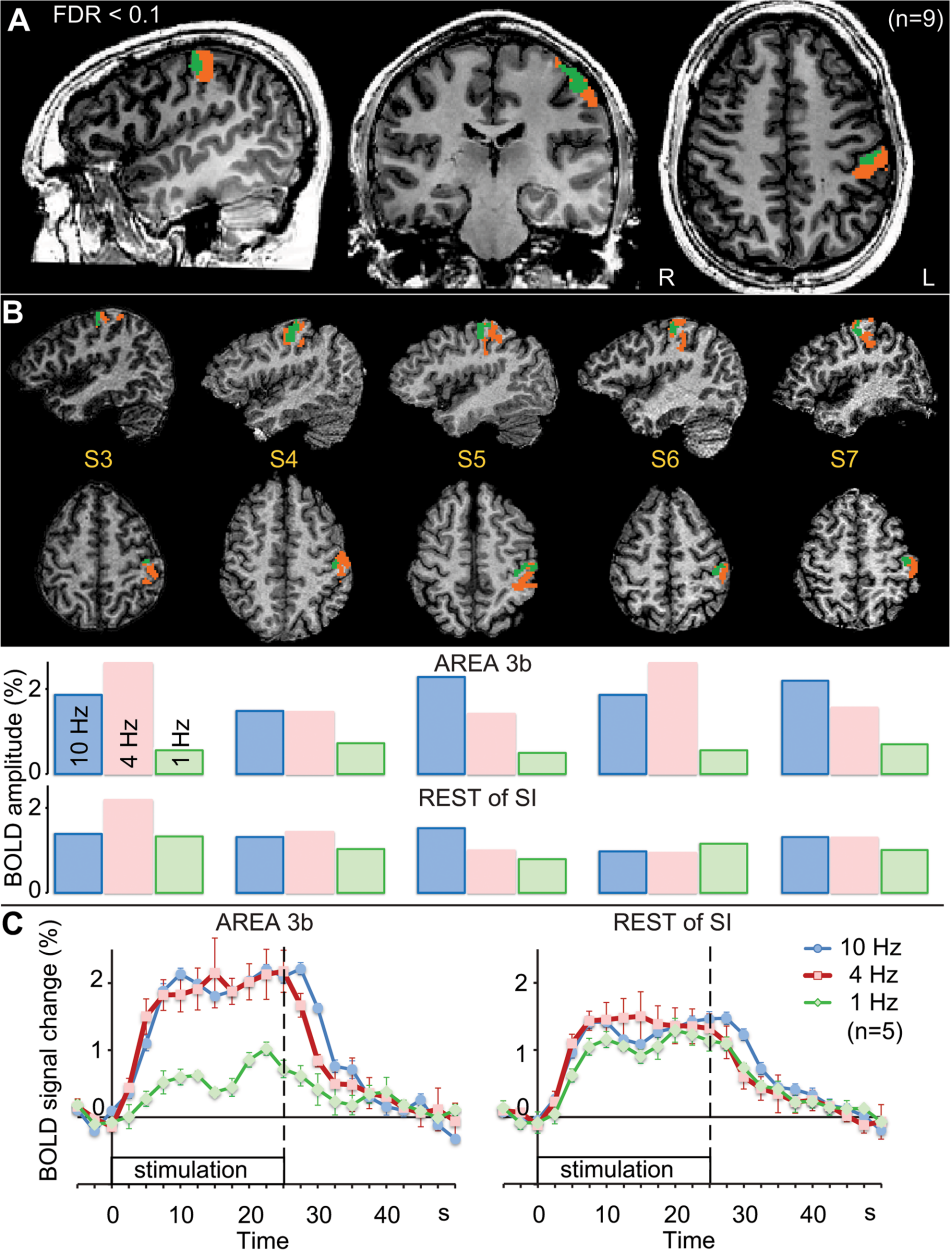
Stimulus-rate sensitive subcluster within SI. A. Group results (n = 9): The statistical map overlaid onto one subject’s Talairach-normalized anatomical images. Contrast ([1Hz] + [4Hz] + [10Hz] – [baseline]) at false-discovery rate q(FDR) < 0.1 was used to define SI activation cluster (orange + green colors). The green color demarcates the stimulus-rate sensitive subcluster in SI obtained in the conjunction of the contrasts: [4 Hz − 1 Hz] > 0 and [10 Hz − 1 Hz] > 0 (presumably area 3b; see Results). B. Individual subjects’ results: Stimulus-rate sensitive subclusters within SI corresponded to the conventional location of area 3b in 5 out of 9 subjects. The stimulus-rate sensitive cluster “AREA 3b” is marked with green color, while the “REST of SI” cluster with orange. The SI cluster was defined at q(FDR) < 0. 1, and in the subsequent search for stimulus-rate sensitive voxels within that cluster, the statistical threshold was q(FDR) < 0.1 (see Methods for details). The bar graphs show for each subject and each SR the BOLD-response amplitudes in “AREA 3b” (top row) and in the “REST of SI” (bottom row) clusters (estimated as the average % BOLD response across the stimulus block, starting 5 s after stimulus onset and ending 5 s after stimulus offset). C. Response time courses: The insets show the BOLD responses (mean ± SEM) for the stimulus-rate sensitive cluster “AREA 3b” (on the left) and for the rest of the SI cluster (on the right). The data correspond to the average of the individual clusters shown in panel B (n = 5).

Data analysis using the main contrast revealed prominent activations (positive BOLD responses) in the contralateral (left) SI cortex and bilaterally in the parietal operculum (SII region). Based on cluster-level time courses, none of these clusters featured statistically significant distinction between the stimulation frequencies (for details of the activation patterns, see [18]). We used the contralateral rolandic cluster obtained from this contrast (thresholded at q(FDR) < 0.1 both in group and individual subject analyses; green + orange cluster in Fig 2A and 2B) to define the extent of the activated SI cortex for the subsequent analysis.

### Stimulus-rate sensitivity within SI cortex

The SR-sensitive conjunction of the contrasts: [4 Hz − 1 Hz] > 0 and [10 Hz − 1 Hz] >0 (FDR < 0.1 within the SI cluster; t = 3.53; 9 subjects, 8 degrees of freedom) in the group data revealed a subcluster within the contralateral rolandic cluster. This SR-sensitive subarea occupied the anterior-ventral part of the SI cluster (green cluster in Fig 2A), which corresponds to the position of area 3b in the rostral bank of the postcentral gyrus, in agreement with cytoarchitectonic population maps of area 3b (see Fig 6 in [23]). The location of the activation is also in line with fMRI mapping of the area 3b representation of fingertips (Fig 1 in [24]).

Analysis of individual subjects’ data revealed an SR-sensitive subcluster within the SI cortex in 5 (out of 9) subjects (Fig 2B), in whom the SR-sensitive subcluster consistently encompassed the postcentral gyrus and the conventional location of area 3b in the posterior bank of the central sulcus (similar to the group map). To search for other SR-sensitive clusters, we performed an additional whole-brain analysis at the group level by looking at the conjunction of contrasts: i) ([10 Hz + 4 Hz + 1 Hz] > 0 and ii) the SR-sensitive conjunction of the contrasts ([4 Hz − 1 Hz] > 0 and [10 Hz − 1 Hz] >0). This analysis revealed only one cerebral activation cluster (corresponding to our “AREA 3b” cluster) in the threshold range from t = 5.3 to t = 3.3 (8 degrees of freedom; corresponds to p_uncorr_ = 0.0008 and 0.01; cluster-size threshold set to ten normalized voxels). This analysis precluded speculations about SR sensitivity in other brain regions (cf. [10]), and while it demonstrated SR-sensitive segregation of SI, we preferred the within-ROI approach which made the analysis procedure coherent at both group and individual levels.

The lower part of Fig 2B shows the average amplitude of BOLD response in putative area 3b and the rest of SI clusters for each SR and in each subject (estimated as the average % BOLD signal change across the stimulus block, starting 5 s after stimulus onset and ending 5 s after stimulus offset). Fig 2C depicts the time courses of activation in the SR-sensitive subarea (”AREA 3b” on the left) and the rest of the SI cluster (”REST of SI” on the right), averaged for the individual clusters shown in Fig 2B (n = 5).

### Comparison with cytoarchitectonic population maps

We also compared our results with the cytoarchitectonic probabilistic population maps [2, 23]. First, the Talairach coordinates of the SR-sensitive subcluster’s peak (–47, –23, 49) were transformed into SPM-MNI coordinates using the *tal2icbm_spm* function incorporating the Lancaster transform (expected error within 1–3mm; [25]). The resulting coordinates (–49, -18, 53) were input to the ANATOMY SPM-toolbox v1.8 (http://www.fz-juelich.de/inm/inm-1/DE/Forschung/_docs/SPMAnatomyToolbox/SPMAnatomyToolbox_node.html; [26, 27]) which estimated that the functional subcluster could be ascribed to area 3b with 40% probability, to area 1 with 80% and to area 4 with 20% probability. The “erroneous” cumulative probability of 140% might be due to several reasons such as different sets of post-mortem brains used for the mapping of neighboring cytoarchitectonic areas and, in particular, partial volume effects, and interpolation and rounding necessitated in the course of constructing the cytoarchitectonic probabilistic population maps in standard space (Simon Eickhoff, personal communication).

## Discussion

The present study demonstrated that the SR sensitivity of the BOLD response differs in a subarea of SI from the rest of the SI cortex and thereby could be used to identify and functionally segregate this region that we consider to correspond to the cytoarchitectonic area 3b. Specifically, the SR-sensitive contrast might serve as a functional localizer of area 3b in studies of the cortical somatosensory network. This result extends previous studies that suggested segregation of the whole SI cortex by means of its SR-dependence [7, 8].

### SR effect in fMRI and electrophysiological recordings

Our results showing BOLD signal increase as a function of increasing SR corroborate previous fMRI findings [7–9]. However, numerous magnetoencephalography (MEG; [9, 28–32]) and electroencephalography (EEG; for a review, see [33]) studies have demonstrated the general tendency for the amplitudes of the SI responses to decrease, and not increase, with higher SRs.

We have demonstrated earlier [34] that one of the reasons for this apparent discrepancy is the traditional way in which electrophysiological and hemodynamic signals are compared. Typically, the peak amplitudes of MEG or EEG responses to individual stimuli within a block are compared with the peak or mean fMRI signal in response to the entire block. However, the fMRI signal reflects the hemodynamic response to a *succession* of neuronal events elicited by the block of stimuli.

To relate the different measurements, one should rather compare the hemodynamic response with an estimate of the total neural activity elicited over the stimulus block. In rat SI cortex, for example, the amplitude of somatosensory evoked potential (SEP) multiplied by the number of stimuli within the stimulation block correlates with the amplitude of cerebral-blood-flow response [35]. This finding is consistent with a human fMRI–EEG study demonstrating a linear coupling between the amplitude of BOLD response in human SI and the SEP amplitude in an experiment in which the intensity of electrical stimuli was modulated [36].

In our previous study [34], we showed that by utilizing the energy density of the MEG source waveform over the whole stimulation block, the MEG signals predict the SR-dependent changes of the BOLD signal in area 3b,as delineated in the present study. Specifically, while the amplitude of the 50-ms MEG response decreased with increasing SR, the energy density increased from 1- to 4-Hz SR, with no further change at 10 Hz; furthermore, these energy density waveforms predicted the SR-dependent changes of the BOLD responses. Thus, there is a clear correspondence between fMRI and electrophysiological data [34].

But why would one observe SR-sensitive BOLD only in area 3b and not in other SI subareas? Areas 1 and 2 are downstream from area 3b in the tactile-processing sequence [16, 17], and they therefore reflect more polysynaptic processing. Consequently, the decrease of neuronal responses (as reflected in MEG/EEG) as a function of increasing SR is expected to be more prominent in areas 1 and 2 than in area 3b. As discussed above, the increase of the number of stimuli within a block (e.g. by a factor of 10 when the SR increases from 1 to 10 Hz) can overcompensate for the *decrease* of neuronal responses from say 1 to 10 Hz so that the corresponding BOLD response even *increases* as a function of SR in area 3b [34]. However, apparently due to the more prominent decrease of the neuronal responses in areas 1 and 2, the BOLD response amplitude—proportional to the total energy density of neuronal responses times the number of stimuli—remained about the same at all investigated SRs in the rest of SI.

### Functional segregation of SI subareas

Qualitatively, the observed inter-individual variability of anatomical location, as well as of the extent of “AREA 3b” clusters in our study is consistent with the variability observed in cytoarchitectonic population maps of area 3b [23]. An additional quantitative comparison with the cytoarchitectonic population maps failed to give a clear preference to area 3b. We do not consider this result surprising given the great inter-individual variability in the location of area 3b. Moreover, the numerous spatial transforms required inevitably deteriorate the spatial precision [27].

Based on the geometry and position of the observed subclusters, especially at the group level (anterior-ventral part of the SI cluster presumably encompassing areas 3b, 1 and 2), one can, however, conclude that area 3b features SR-sensitive BOLD responses. Of course, the extent of any fMRI activation is illusory as it depends on the signal/contrast-to-noise ratio and on the threshold applied; thus, it is not possible to completely rule out involvement of the neighboring area 1. However, stimulation of the fingertips should facilitate discrimination of area 3b and area 1 activations because the main activation clusters are expected near the border of areas 3a/3b and areas 1/2 but not at area 3b/1 border, as has been demonstrated in previous human fMRI studies [24, 37]. If SR sensitivity were a common feature of both areas 3b and 1, one would expect the SR-sensitive cluster to also encompass the rostral and superior part of the SI cluster. SR sensitivity appeared, however, limited to the anterior-ventral subcluster, thereby suggesting involvement of area 3b only.

Four adjacent mirror maps of index-finger representations have been recently demonstrated in individual humans using high-spatial-resolution fMRI recording at 7 T [37]. Although that study employed vibrotactile stimuli (*vs* pneumotactile stimuli in our study), the success ratio of obtaining multiple distinct maps was 4/6 (4 out of 6 subjects), which is in line with the success ratio of 5/9 in our study. The failure of both methods in part of the subjects might be related to the lack of typical functional segregation of area 3b. At the same time, the similar success ratio despite considerable difference in spatial resolution and magnet field strength (3.13 × 3.13 × 4 mm^3^ at 3T *vs*. 1.25 × 1.25 × 1.3 mm^3^ at 7T) substantiates robustness of SR sensitivity segregation.

Notwithstanding the method's robustness, the borders between all subareas of SI cannot be accurately identified by only looking at the SR-sensitivity of the functional images; instead, some additional functional features would be needed. For example, one might utilize the reversal of the somatotopic representation maps at the border of SI subareas [24, 37], Additionally one could employ other features such smearing of the somatotopy [38], overlap of finger representations [39–41], and suppressive interaction of tactile inputs [42] that build up in the rostro-caudal direction through areas 3b, 1, and 2. All these differences, along with the SR sensitivity, could be combined to develop a robust test battery to sharpen the noninvasive functional segregation of the human SI cortex at both 3T and 7T, thereby bringing studies of the somatosensory cortex closer to the now well-established functional mapping techniques of the human visual cortices [5].
